# Bridging Nano-Interface Interactions and Organ-Specific Toxicity: A Review of Machine Learning for Nanomaterials Risk Assessment

**DOI:** 10.3390/molecules31183293

**Published:** 2026-09-17

**Authors:** Wei Li, Yanfang Liu, Tianqin Wang, Jiana Meng, Yang Huang, Jiajun Ma, Hongwu Zhang

**Affiliations:** 1 School of Chemistry and Chemical Engineering, Ludong University, Yantai 264025, China; gemo4893@gmail.com (W.L.); 1wangtianqin1@gmail.com (T.W.); 15865768679@163.com (J.M.); hwzhang@iue.ac.cn (H.Z.); 2 School of Statistics, Shandong Technology and Business University, Yantai 264005, China; liuyanfang22@126.com; 3 Department of Chemical and Biomolecular Engineering, The Hong Kong University of Science and Technology, Hong Kong 999077, China; jmaby@connect.ust.hk

**Keywords:** ML, emerging pollutants, health effect, nanomaterials, predictive model, safe-and-sustainable-by-design (SSbD)

## Abstract

Risk assessment of engineered nanomaterials (ENMs) is essential for protecting human health and the environment. Traditional hazard assessments rely primarily on in vivo testing, which faces technical challenges in extrapolation validity, ethical dilemmas, and high costs. Machine learning (ML) models offer alternative approaches that are aligned with the 3R principles (Replacement, Reduction, and Refinement) for reducing animal use. ML methods help address the economic, ethical, and temporal limitations of traditional nanotoxicology while advancing mechanistic understanding. This review presents a cross-scale framework integrating nano–bio/nano–environmental interfaces, organ-specific toxicity, in vitro-to-in vivo extrapolation (IVIVE), interpretable ML, and regulatory translation. Future directions include building comprehensive databases to replace sparse literature data, developing ML models that bridge in vitro and in vivo nanotoxicity, incorporating co-exposure scenarios of nanomaterials and chemicals, and further exploring protein/lipid corona formation and structures.

## 1. Background

With the widespread application of nanomaterials in fields such as medicine, chemicals, and the environment, their potential toxicity has garnered increasing attention. Traditional toxicity assessment methods rely heavily on in vivo experiments, which are associated with high costs, long durations, and ethical concerns. In recent years, machine learning (ML) has emerged as a powerful data analysis tool in the field of nanotoxicology, capable of constructing predictive models from complex, high-dimensional datasets and elucidating the underlying mechanisms of nanomaterial toxicity. This review aims to summarize the current research progress in ML-based nanotoxicity prediction, evaluate its potential and limitations in risk assessment, and explore future directions for development. The necessity of this review arises from a critical mismatch: the number and diversity of engineered nanomaterials (ENMs) are expanding faster than conventional toxicity testing can evaluate them, while regulatory decision-making increasingly requires rapid, mechanistically interpretable, and ethically acceptable evidence. This review provides insights into synthesizing how ML can connect nanomaterial descriptors, nano–bio interface behavior, organ-level adverse outcomes, and environmental co-exposure into an integrated risk-assessment framework.

## 2. Introduction

Studies in nanotoxicology have generated extensive and diverse datasets. The challenge lies in extracting key insights hidden [1] within the vast flow of data. Artificial intelligence (AI) and ML play a pivotal role in transforming nanotoxicity data into critical information, specifically by constructing quantitative nanostructure (physicochemical properties)–toxicity relationships and elucidating toxicity-related molecular mechanisms [2,3,4]. Therefore, the core scientific question is no longer whether ML can predict individual toxicity endpoints, but whether it can convert fragmented nanotoxicity observations into generalizable, mechanism-aware evidence for risk assessment.

In recent years, with the rapid development of nanotechnology, there has been an increasing use of nanomaterials in medicine, energy, the environment, and other fields [5]. However, the extensive utilization of these materials has also given rise to concerns regarding their potential toxicity. In contrast to conventional chemicals, the toxicity of nanomaterials is influenced by a multitude of factors, including their molecular structure, physicochemical properties, and interactions at the nanobiological interface. The behavior of nanomaterials in biological environments, such as the formation of protein crowns, cellular uptake, and immune system response, greatly increases the complexity of their toxicity assessment [6]. Consequently, the development of accurate predictive models for nanotoxicity has emerged as a pivotal challenge in the field of nanotoxicology research. Traditional toxicity assessment methods, which primarily rely on animal experiments and in vitro studies, can provide direct toxicity data. However, they are costly, time-consuming, and raise ethical concerns [7]. Moreover, the diversity and complexity of nanomaterials make it difficult for traditional toxicity assessment methods to fully reflect their toxicity mechanisms [8]. In particular, the behavior of nanomaterials at the bio-interface, such as surface modification, size effects, and biomolecule adsorption, further complicates toxicity assessment [9]. Therefore, the development of new toxicity prediction methods, especially computational models based on ML, has become an important direction in nanotoxicology research [10,11]. This complexity makes nanotoxicity a particularly appropriate field for ML because toxicity does not originate from a single molecular feature but from dynamic interactions among size, shape, surface chemistry, dissolution, corona formation, exposure scenario, and biological context.

ML excels at resolving the unique complexity of nanotoxicology arising from multidimensional nano–bio interfacial coupling. Unlike conventional statistical methods that depend on preset variables and linear correlation assumptions, ML can disentangle non-linear, synergistic relationships between hierarchical nanomaterial physicochemical features and dynamic interfacial biological behaviors, thereby capturing context-dependent toxic responses that are inaccessible to traditional analytical tools [9,11]. Such strengths enable the mechanistic decoding of dynamic nano–bio identity transformation driven by biomolecular corona formation and support reliable prediction of nanotoxicity across diverse exposure and biological scenarios [12].

The challenge in constructing prediction models lies in the fact that the toxicological effects of ENMs are determined by multiple factors, including molecular structure, physicochemical properties, and nano–bio interfacial interactions. At the nano–bio interface, changes in the physicochemical properties and biochemical behavior of ENMs can significantly influence the immune system’s recognition and response, thereby affecting their toxicity [13]. Compared to computational toxicology studies of traditional chemicals, this process greatly increases the complexity of nanotoxicity modeling. This is specifically manifested in the following: First, existing nanotoxicity data are often scattered and incomplete, lacking systematicity and consistency, which limits the construction and validation of ML models [1,14,15]. Second, the toxicity mechanisms of nanomaterials are complex [16], involving multiple biological levels and various toxicity endpoints. How to integrate these multi-source data into a unified model remains an urgent problem to be solved. In addition, ML models generally have poor interpretability, and how to extract meaningful biological mechanisms from these models is also an important direction for future research. These challenges also define the knowledge gap targeted by this review: existing studies often discuss algorithms, datasets, or specific organs separately, whereas risk assessment requires a cross-scale synthesis linking data quality, feature representation, model interpretability, biological plausibility, and regulatory applicability. However, the majority of existing nanotoxicology reviews seldom address these three critical dimensions. Current black-box machine learning algorithms do not provide clear causal mechanistic explanations, and most models oversimplify actual physiological and environmental exposure conditions, thereby diminishing biological relevance. Additionally, inconsistent data standards and the absence of validation metrics impede their acceptance by chemical regulatory authorities. Consequently, this work aims to incorporate a focused discussion of these three aspects in the subsequent sections to address this gap.

ML can play a crucial role in addressing these challenges. Due to its powerful data analysis and fitting capabilities, ML algorithms show great promise in handling complex data patterns [17]. In computer systems, “empirical rules” are often stored in data, and ML refers to a class of algorithms that generate “models” from data. The ML modeling process in computational toxicology consists of five main steps: database construction, data preprocessing, model building, model validation, and mechanistic interpretation. In contrast to conventional statistical methodologies, it possesses the capacity to manage a substantial number of features that exhibit a weak or non-linear association with the predicted endpoint *y*. In the context of ML models, significant information is not encapsulated within a solitary input variable *x_i_*. Consequently, crucial variables cannot be predicted prior to modeling; rather, predictions must be determined through the integration of a specific combination of some features that are unknown prior to modeling. The unique properties of ML make it particularly suitable for solving complex nano–bioeffects problems with multidimensional input feature sets, identifying toxicity mechanisms that cannot be captured by traditional tools. Given that the relationship between toxicity endpoints and feature parameters in complex biological environments is frequently non-linear and multifaceted, the utilization of ML-based toxicity prediction tools is poised to emerge as a pivotal paradigm shift in the domains of toxicity screening and ENM regulation [18]. A thorough examination of the annual number of publications in PubMed discloses that the number of studies in the domain of toxicity prediction by artificial intelligence methods, such as ML, exhibited a marked increase. Notably, there was a nearly fivefold surge in publications between 2014 and 2020 [18]. Future research should focus on facilitating the transition of nanotoxicology from an experiment-driven to a data-driven discipline [19]. A review that explicitly bridges these factors is needed to guide both model development and experimental design. The present review adopts a cross-scale, exposure-to-outcome perspective across ENMs. It links dynamic nano–bio and nano–environmental interfaces (including biomolecular corona formation and interactions with coexisting pollutants) to organ-specific adverse outcomes, IVIVE, interpretable prediction, and Safe-and-Sustainable-by-Design (SSbD) decisions, thereby providing a biologically and environmentally contextualized framework that complements previous model-centered syntheses.

## 3. Literature Search and Study Selection

A prominent multidisciplinary database, Web of Science (https://www.webofscience.com), was utilized for literature mining. The combined retrieval keywords included nanotoxicology, engineered nanomaterials, nanomaterials, machine learning, artificial intelligence, toxicity prediction, nano–bio interaction, QSAR, and QNAR. All retrieved publications were limited to the period from 2010 to 2026, encompassing the major developmental phase of machine learning applications in nanotoxicology. During screening, articles were included if they directly addressed machine learning or related computational approaches for evaluating ENM toxicity and nano–biomacromolecule or nano–pollutant interactions, or provided essential mechanistic evidence relevant to these endpoints. Duplicate records, studies unrelated to nanoscale materials or risk assessment, and reports lacking sufficient methodological detail were excluded.

## 4. ML Models for Predicting Toxicity of Nanomaterials

Computational toxicology tools are essential for increasing throughput, reducing the burden of animal testing, providing details of the toxicity mechanisms, and generating novel hypotheses for risk assessment in nanotoxicity. The ML models for predicting various forms of nanotoxicity are discussed below.

### 4.1. Pulmonary Toxicity

Inhalation of aerosols created during industrial processes is a main route of nanomaterial exposure to humans [20]. Some nanoparticles were found to induce strong immune responses in the lungs via inflammasome and Toll-like receptor activation in vitro [16] and in vivo [21], evidenced by substantial release of pro-inflammatory cytokine (IL-1β). From the perspective of nano safety assessments [22], ML models have been developed for predicting nanomaterials’ lung toxicity and assisting biocompatible material design (Table 1).

**Figure 1:**
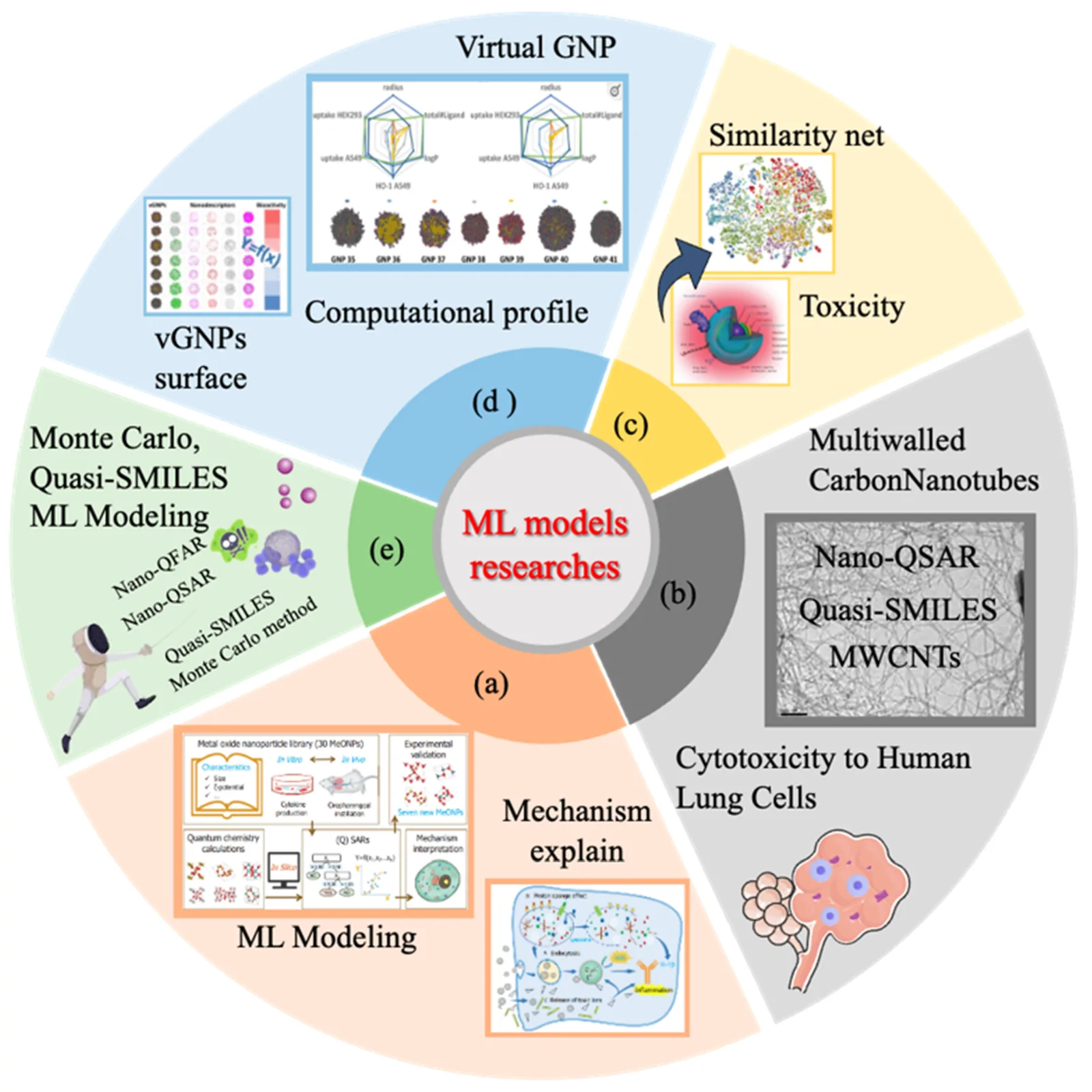
ML models for pulmonary toxicity prediction. (**a**) QSAR models predicting inflammatory effects of metal oxide nanoparticles (MeONPs). (**b**) Quasi-SMILES-based QSAR models for toxicity prediction of 20 multi-walled carbon nanotubes (MWCNTs) in human lung cells. (**c**) QSAR models for toxicity similarity network visualization. (**d**) QNAR model based on surface information of virtual gold nanoparticles (vGNPs). (**e**) Monte Carlo Nano-QSPR/Nano-QSAR models using Quasi-SMILES for nanomaterial toxicity prediction. Quasi-SMILES: a simplified linear coding descriptor extended from standard SMILES molecular notation [26,27].

As shown in Figure 1, Oh et al. [23] established cellular toxicity models to predict the toxicity in epithelial cells and fibroblast cells in the lung, induced by 17 quantum dots using surface properties, diameters of quantum dots, assay types, and exposure times. ML models were developed to predict the cytotoxicity of 20 different types of multiwalled carbon nanotubes (MWCNTs) to human lung cells [24]. The model showed that MWCNTs with several properties (diameter, 12–74 nm; length, 0.19–20.25 μm; surface area, 11.3–380.0 m^2^/g; and dose, 0–200 ppm) have potential toxicity for human lung cells. Wang et al. [25] integrated 240 data points of 30 metal oxide nanoparticles (MeONPs) and established a high-precision prediction model using the synthetic minority oversampling technique for regression (SMOTER) to solve the data imbalance problem.

A combined experimental and computational study has been performed to estimate the acute lung toxicity of metal oxide nanoparticles [28]. Predictive models were established for predicting the median lethal concentration (*LC*_50_) of metal oxide nanoparticles to human lung adenocarcinoma (A549) cells by considering the influence of particle size and zeta potential on the cytotoxicity. The models showed that the toxic mechanism responsible for the cytotoxicity of metal oxide nanoparticles to A549 cells was related to reactive oxygen species (ROS) release [28,29]. The lung inflammatory potential of ENMs has been predicted since nanoparticles have exhibited subtle effects beyond merely killing cells. ML models were built to predict the lung inflammatory potential of metal oxide nanoparticles [30]. A comprehensive dataset of 30 metal oxide nanoparticles was built to screen IL-1β release in a macrophage-like myeloid cell line, THP-1 cells. Prediction models with predictive accuracy (ACC) exceeding 90% were developed for inflammatory potential. Electronegativity, ζ-potential, and cation charge were identified as three key properties responsible for the inflammatory effects of metal oxide nanoparticles.

Given the complexity of the lung and the complicated properties of nanoparticles, conventional quantitative structure–activity relationships (QSARs) exhibit significant limitations in precisely predicting the in vivo lung toxicity and organ burden of nanoparticles. The development of ML provides solutions for predicting the complicated toxicity of nanoparticles in the lung. Yu et al. [31] proposed a tree-based random forest feature importance and feature interaction network analysis framework (TBRFA) and accurately predicted the pulmonary immune responses and lung burden of nanoparticles in a mouse model [31]. The correlation coefficients of all training sets exceed 0.9, and half of the test sets exceed 0.75. Although efforts have been made to predict in vivo nanotoxicity in the lung, ML models that decode relationships between in vitro and in vivo nanotoxicity are still inadequate and should be developed in the future [32]. Cao et al. [33] further adopted random forest classification to correlate in vitro nano–bio interface signatures with in vivo nanoparticle-triggered lung fibrosis, identifying seven predictive descriptors and validating the feasibility of fibrotic risk prediction. Recent studies have employed in vitro-to-in vivo extrapolation (IVIVE) approaches for nanoparticle-induced pulmonary toxicity, aiming to elucidate the relationships between in vitro endpoints and in vivo outcomes by integrating multi-source, multidimensional physicochemical, and biological data through advanced data mining techniques [34].

The pulmonary system illustrates why ML-based nanotoxicity prediction is urgently needed. It is the primary exposure route for many occupational and environmental ENMs, yet lung toxicity involves acute cytotoxicity, inflammation, immune activation, fibrosis, and particle burden that cannot be captured by a single assay. The innovative value of ML lies in its ability to integrate physicochemical descriptors, in vitro biomarkers, exposure conditions, and in vivo outcomes to build IVIVE-oriented predictive frameworks.

**Table 1:** ML models for predicting the lung toxicity of nanomaterials.

Nanomaterials	Organism/Organ/Cell	Key Descriptors	Endpoint	Reference
17 quantum dots	Epithelial cells Fibroblast cells	Shell, ligand, surface modifications, diameter, assay type, and exposure time	*LC*_50_	[23]
21 MeONPs	A549 cells	Particle size and zeta potential	*LC*_50_	[35]
34 gold nanoparticles	A549 and HEK293 cells	Hydrophobic potential	Cellular uptake	[1]
20 MWCNTs	human lung cells (BEAS-2B, 16HBE14o-, WI-38, and HBE)	Diameter, length, surface area, and dose	CV	[24]
30 MeONPs	THP-1 cells	Electronegativity, zeta potential, and cation charge	Inflammatory potential	[30]
1620 samples	Mouse lung	Exposure dose and recovery duration	Immune responses and nanomaterial burden in lung	[31]
27 MeONPs	THP-1 cells	IL-1β, PSF, ζ-potential	lung fibrosis	[34]
50 MeONPs	THP-1 cells, BEAS-2B cells, Mouse lungs	IL-1β, NADH, TGF-β1, Dissolution in PSF, ζ-potential, Hydrodynamic size	Lung fibrosis	[33]

MWCNTs multiwalled carbon nanotubes; CV: cell viability %; EC_50_: concentration for 50% of maximal effect; LC_50_: median lethal concentration.

Expanding the analysis beyond the lung is necessary because systemic translocation determines long-term health risks and can reveal toxicity mechanisms that are invisible in respiratory models alone. After entering the human body, nanomaterials will cross the blood–air barrier and encounter various target organs and systems (liver, gastrointestinal tract, nervous system, reproductive system, etc.). Previous studies reported that various types of ENMs are able to pass biological barriers and exert toxic effects on important tissues [36]. Studies in rats have shown that ENMs can translocate to interstitial sites in the respiratory tract, as well as to extrapulmonary organs such as the liver within 4 to 24 h postexposure [37,38]. It is thus necessary to develop ML models for predicting nanotoxicity in these organs/systems.

### 4.2. Nanotoxicity in Other Organs

Studies to date have pointed out that ENMs can cross the epithelial barrier, enter the bloodstream, and ultimately distribute throughout the organism [39,40,41]. The toxicity of nanoparticles in organs other than the lung is also the focus of research. Several studies have shown that inhaled nanoparticles also have adverse effects on other non-respiratory organs, such as hepatotoxicity, nephrotoxicity, reproductive toxicity, neurotoxicity, etc. [42,43,44].

**Figure 2:**
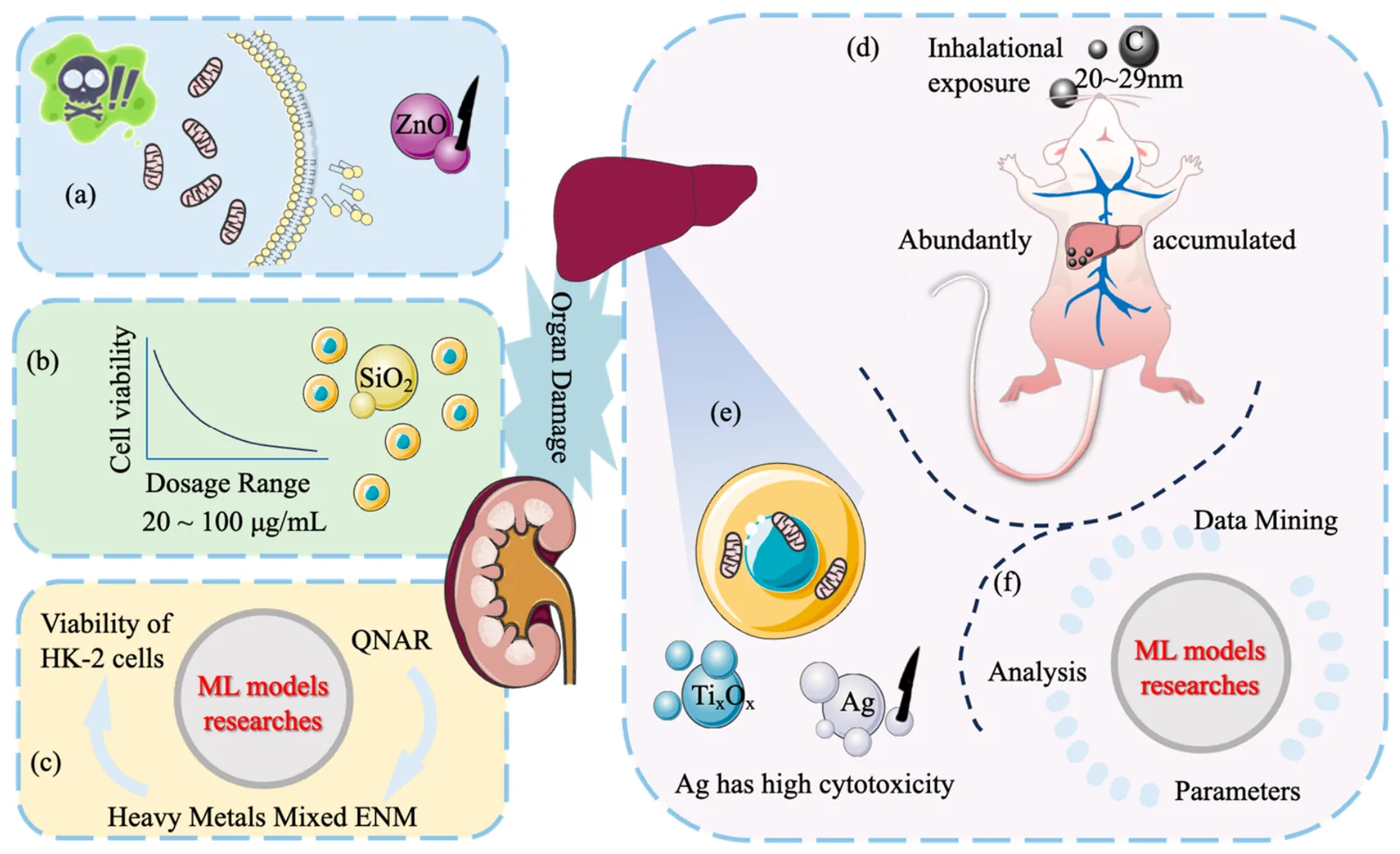
ML models for hepatotoxicity and nephrotoxicity of ENMs. (**a**) ZnO NPs induce nephrotoxicity in rats via mitochondrial and membrane damage. (**b**) Dose-dependent cytotoxicity of SiO_2_ NPs in HEK293 cells (20–100 μg/mL). (**c**) QNAR model prediction of TiO_2_ NPs toxicity in HK-2 cells. (**d**) Rapid hepatic accumulation of 20–29 nm carbon NPs after inhalation in rats. (**e**) Comparative hepatotoxicity of TiO_2_ and Ag NPs in BRL 3A cells; Ag > TiO_2_. (**f**) Classification models for ENM hepatotoxicity prediction.

Among them, the most common accumulation target tissues are the liver and kidney. In addition, it has been reported that ENMs can cause damage to various tissues and organs, such as inflammatory reaction in the liver, atrophy, and necrosis of renal tubules, and hyperplasia of renal interstitial fibrous tissue [45,46,47]. The liver is the main detoxification organ of the human body, which participates in the catabolism, inactivation, and excretion of many exogenous and endogenous substances. Liver function can be impaired when exposed to ENMs acutely or chronically. Oberdöster et al. [48] reported that carbon nanoparticles in the size range of 20–29 nm were abundantly accumulated in rat liver during 30 min of inhalational exposure [48] (Figure 2d). This points to rapid translocation of nanoparticles in the liver. Kakakhel et al. [49] provided direct evidence of significant accumulation and toxic effects of silver nanoparticles (AgNPs) in the liver of living organisms. This accumulation was associated with hepatic tissue damage and induced alterations in key biomarkers of oxidative stress responses.

Hussain et al. [50] compared the toxicity of silver and titanium oxide nanoparticles by using the rat liver cell line BRL 3A. Cytotoxicity, mitochondrial dysfunction, and oxidative stress were carefully studied as endpoints of hepatotoxicity. The work investigated that silver has higher cytotoxicity among them. Interestingly, Sahu et al. also observed the cytotoxicity of nano-silver in human liver HepG2 and colonic Caco2 cells [51].

One study proposed a toxicity classification model for seven different oxide nanomaterials, including L-02 cells and Chang-Liver cells associated with liver injury. Based on useful data obtained from the literature, Choi et al. built classification models of four different algorithms by using generalized linear models, support vector machines, random forests, and neural networks, and compared their performance to obtain the optimal model. Among them, building models using neural networks has the best predictive performance. Dose, enthalpy of formation, exposure time, and hydrodynamic size were determined as the four most important factors in the relative attribute importance analysis [52]. Similarly, Wang et al. [25] found that concentration, exposure time, and hydrodynamic size are also important factors in the cytotoxicity of nanoparticles.

The kidney is another common target organ of ENM toxicity. It has been reported that the kidney is an important target organ of toxicity and the main organ in the process of ENM removal. Yan et al. [53] evaluated the nephrotoxicity of ZnO nanoparticles in rats by analyzing the urine and kidney tissue of rats treated with ZnO nanoparticles. This study showed that rat kidney mitochondria and cell membranes were damaged in the presence of ZnO nanoparticles, resulting in nephrotoxicity [53]. Kim et al. [54] further employed lipid pathway enrichment analysis (LIPEA) and found that ZnO nanoparticles induced cytotoxicity and modulated lipid species in human renal epithelial cells. Another interesting study, in order to explore the effect of nanoparticles on nephrotoxicity and its mechanism, is Wang et al. [39], who compared the effects of 20 nm and 50 nm silica nanoparticles on human embryonic kidney (HEK 293) cells as an in vitro model. The study compared the control group and the experimental group, which was exposed to silica conditions; the study observed cell viability, mitochondrial function, cell morphology, reactive oxygen species (ROS), glutathione (GSH), thiobarbituric acid reactive substances (TBARS), cell cycle, and apoptosis. The results showed that the activity of HEK293 cells decreased in a dose-dependent manner when exposed to SiO_2_ nanoparticles in the dose range of 20~100 μg/mL. Figure 2 summarizes the ML models for hepatotoxicity and nephrotoxicity of ENMs.

Based on the above study, a prediction model for cytotoxicity of HEK293 cells exposed to 20 and 50 nm silica NPs was established by Manganelli et al. [55] through using a quasi-SMILES descriptor. Model establishment relies on a mathematical function between cell viability, ENM size (20 nm, 50 nm), ENM concentration (25~200 μg/mL), and exposure time (0~48 h). All data were randomly divided into three datasets for training, calibration, and verification. Then, CORAL software (http://www.insilico.eu/coral/) was used for calculations. The toxicity was assessed by analyzing the cell viability of human embryonic kidney cells using the 3-[4,5-dimethylthiazole-2-yl]-2,5-diphenyltetrazolium bromide (MTT) assay. The quality of the model is satisfactory, and the R (2) value of the best model is more than 0.68 [55]. Yuan et al. [56] developed a QSAR in the nanomaterial field (QNAR) model to predict the cell viability of HK-2 cells exposed to nano TiO2. HK-2 cells were exposed to four groups of mixtures containing heavy metals and ENMs, and cell viability and ROS were measured to study nephrotoxicity. The QNAR model was established using Multiple Partial Least Squares Regression (PLS) and Random Forest Regression (RF). The models accurately predicted the viability of HK-2 cells after exposure. Among them, the RF model showed higher stability and higher precision in assessing nephrotoxicity. Recent advances have also been made in algorithmic innovation. Pandey et al. [57] integrated quantitative read-across (qRA) into QSAR model development to construct a hybrid predictive model for acute inhalation toxicity. Utilizing the ICE database (https://ice.ntp.niehs.nih.gov/DATASETDESCRIPTION, accessed on 13 September 2026), which includes LC50 values for 729 organic compounds, qRA assessed structural similarity based on Euclidean distance across 18 feature parameters. Compared to traditional QSAR models, the hybrid approach reduced the number of descriptors by 70% (7 vs. 24) while improving predictive performance by 13.5%, achieving a Q2 value of 0.605 and a mean absolute error (MAE) of 0.568.

**Figure 3:**
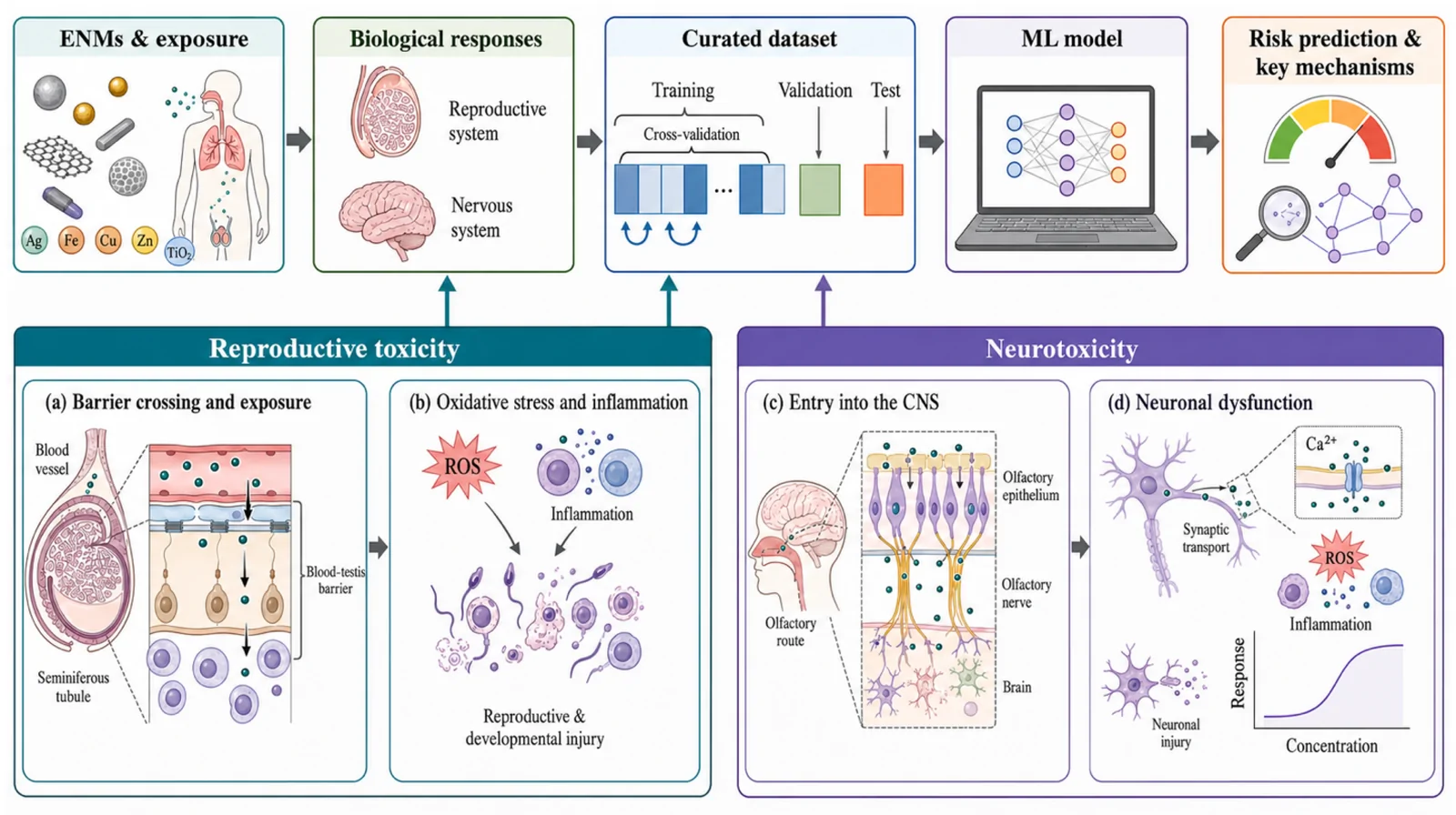
Effects of ENMs on the nervous and reproductive systems. (**a**) Barrier crossing and exposure: ENMs penetrate the blood-testis barrier and reach the seminiferous tubule. (**b**) Oxidative stress and inflammation: ENMs trigger ROS and inflammation to induce reproductive and developmental damage. (**c**) Entry into the CNS: ENMs enter the central nervous system via the olfactory pathway. (**d**) Neuronal dysfunction: ENMs disturb calcium signaling and synaptic transport, causing concentration-dependent neuronal injury. The upper schematic presents the research pipeline from ENM exposure to ML-based risk prediction.

At present, there are few reports focusing on the nephrotoxicity of ENMs. However, the results of these studies suggest that ENMs can induce nephrotoxicity [58,59,60,61]. Therefore, it is necessary to establish ML models to predict the effects of ENMs on hepatotoxicity and nephrotoxicity. ENMs can reach and accumulate in secondary target organs during translocation across biological barriers, where they may induce adverse biological reactions [40]. Figure 3 summarizes the effects of ENMs on the nervous and reproductive systems.

Currently, ENMs have posed a threat to the fragile reproductive system with continuous exposure to ENMs [62]. The discussion of the reproductive toxicity of ENMs and their mechanisms is critical for the development of nanomaterial risk assessments. Past studies have shown that ENMs can not only directly lead to reproductive toxicity, but also indirectly affect developmental toxicity [63,64,65]. There is some clear evidence that reproductive and developmental toxicity is attributable to oxidative stress and inflammation [66].

The small size of ENMs allows them to pass through the blood–testis barrier (BTB), causing them to accumulate in reproductive organs [67]. A study reported that the male reproductive system can be damaged by TiO_2_-ENMs due to the induction of inflammation, cytotoxicity, and changes in gene expression through ENMs passing through the BTB [68].

ML models for predicting the interactions between ENMs and the reproductive system have been developed [69,70,71]. The embryonic zebrafish (*Danio rerio*) is gradually considered as an in vivo test model related to human safety. Robinson et al. built an ML model to predict the toxicity of metal oxide nanomaterials to embryonic zebrafish. The results showed that nano metal-oxides could cause fatal damage to embryonic zebrafish within 24 h after fertilization. In addition, they also cause harmful effects on embryonic zebrafish within 24~120 h after fertilization or at concentrations below 250 ppm [72]. Based on the Biological-Interactions Knowledgebase (http://nbi.oregonstate.edu/, accessed 5 June 2021), models were built using a dataset that included 44 different metal oxide-like nanomaterials. Different modeling methods are also compared on this dataset via nested cross-validation. Multiple descriptors representing the composition of core, shell, and surface functional groups and particle characteristics were used to model the two lethal endpoints, and the results would be useful for the prediction of the reproductive toxicity of ENMs. In Ban’s work, they performed a meta-analysis using data from similar animal studies and predicted the reproductive toxicity of nanomaterials [69]. Herein, the priority factors determining reproductive toxicity were screened from highly heterogeneous data by Ban et al. through relevant literature data and a random forest model, where 10 factors of more than 18 different ENMs were discussed. Among them, the elements contained in ENMs and exposure routes affected the accumulation of ENMs. Additionally, ENM types and toxicity indicators contribute greatly to the reproductive toxicity of various ENMs. Compared with ENMs containing precious elements (rare metallic elements such as gold, silver, and platinum-group metals), ENMs containing major elements accumulate more in rats and have lower toxicity. The combination of a random forest model and similarity network analysis made it possible to reveal the multidimensional factor-toxicity dependencies. Then, we can predict the reproductive toxicity of various ENMs. Notably, Fu et al. [73] reviewed recent advancements in nanotechnology from the unique perspective of women’s reproductive health. Their work highlights the diverse impacts of ENMs across different reproductive stages—including preconception, early pregnancy, mid-pregnancy, and late pregnancy—where ENM exposure has been associated with various adverse health outcomes. However, the authors also emphasize that through the application of advanced nano-enabled diagnostic platforms (e.g., hormone sensors, cell-based detection chips) and therapeutic strategies such as co-delivery systems for nanomedicines, ENMs may conversely play a protective role in safeguarding maternal and fetal health.

ENMs can enter the olfactory bulb from the olfactory epithelial cells in the nasal cavity along the olfactory neurons, so as to reach the brain directly [74,75]. If they can break through the synapse, then they could potentially enter distant structures within the brain. Therefore, ENMs can enter the central nervous system and affect neuronal function [76]. Now, multiple models both in vivo and in vitro have been developed to assess the neurotoxicity of ENMs, and the related mechanisms involved in oxidative stress, inflammation, DNA damage, and cell death have also been investigated [77,78].

In the study by Larner et al., the rat PC-12 neuron-like cell line was used to evaluate the toxicity caused by five nanoparticles: single-walled nanoparticles (SWNTs), fullerene, cadmium selenium quantum dots, carbon black, and dye-doped silica nanospheres. Cell viability assays, cell morphology analyses, and αII-spectrin breakdown products (SBDP) were used as indicators of cytotoxicity for in-depth analysis. The results showed that exposure to higher concentrations (100 µg/mL) of SWNT, carbon black, and fullerene increased the formation of SBDP150/145, cell membrane contraction, and cytoplasmic vacuole formation. In addition, differentiated PC-12 cells seem to be more sensitive to the cytotoxicity of ENMs [79]. Increased cytoplasmic calcium levels and impaired sodium channel functional properties in rat primary cultured hippocampal neurons in the presence of CdSe nanoparticles demonstrate that ENMs are neurotoxic. All biological responses in the central nervous system are directly or indirectly regulated by calcium ions. The instantaneous increase of calcium concentration is unfavorable to each neuron, and more importantly, apoptosis occurs because of a sustained increase in cytoplasmic calcium levels [80].

In the work by Marvin et al., a Bayesian network (BN) was established to evaluate the comprehensive risk based on eight different biological effects (neurological, cardiopulmonary, immunological, inflammation, genotoxicity, and neurotoxicity). The aforementioned BN is composed of different physicochemical properties, biological effects, and exposure methods of ENMs. Fibrosis and cytotoxicity were studied by using p-chem characteristics and information about the type of study (in vitro or in vivo). The BN constructed above has good predictive ability for the effects of ENMs on neurotoxicity and inflammation, etc. [81].

The rapid expansion of ML technology facilitates the development of new evaluation methods for the nanotoxicity of ENMs [17,19]. Furxhi et al. developed an ML classification model for different ENMs regarding nanotoxicity based on data from multiple studies, including cell type, cell origin, toxicity assay method, ENM type, physicochemical properties, and exposure parameters (pathway, time, and concentration) [19].

Hou et al. [82] developed a combinatorial biophysical cue array platform based on dynamic laser interference lithography to systematically investigate the effects of nanomaterial surface topography on macrophage polarization and inflammatory responses. By constructing a high-throughput screening system encompassing over one million distinct nanotopographical patterns and integrating an ML model based on Gaussian process regression, the research team successfully identified key nanotopographical features that induce either M1 (pro-inflammatory) or M2 (anti-inflammatory) macrophage polarization.

As summarized in Table 2, these studies indicate that nanotoxicity is governed by interacting theoretical descriptors, physicochemical properties, and exposure conditions. Computational models can reduce experimental workload, support high-throughput hazard screening, identify major toxicity drivers, and predict pulmonary, hepatic, renal, reproductive, and neurological endpoints. These capabilities provide a quantitative basis for prioritizing confirmatory experiments and informing safer and more sustainable material design. Hepatic and renal studies more often combine exposure variables with formation energy, hydrodynamic size, dissolution, or oxidative stress-related measurements, whereas reproductive and neurological models rely more heavily on material composition, exposure route, concentration, surface coating, and organ- or cell-specific responses. Random forest and related structured data methods facilitate descriptor rankings in small heterogeneous datasets, while Bayesian networks can integrate multiple endpoints and exposure factors [83]. Nevertheless, most studies remain restricted to a single cell type, species, organ, or material class, and only a few provide study-wise or material-wise external validation. Consequently, their utility as reliable surrogate data for regulatory safety decisions is considerably weakened [84].

## 5. ML Models for Predicting Nano–Biomacromolecule/Nano–Pollutant Interactions

**Figure 4:**
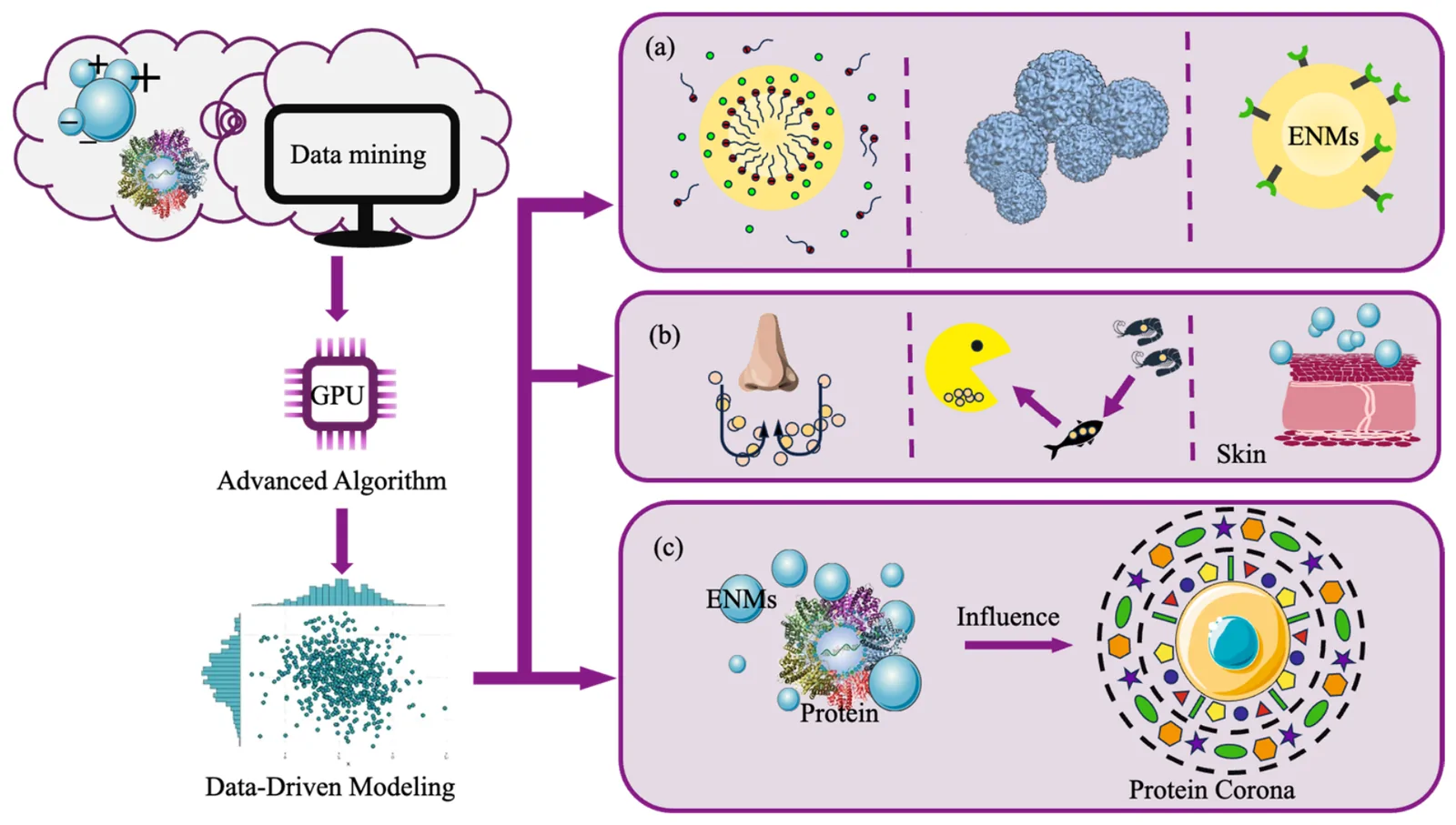
Interaction of ENMs with biomolecules in the human body: (**a**) ENMs exhibit high surface reactivity, large specific surface areas, abundant adsorption sites, and unique physicochemical properties [86]. (**b**) ENMs can enter organisms via incidental routes such as inhalation, ingestion, dermal contact, and food chain accumulation, or through intentional means such as medical diagnostics [88]. (**c**) Protein properties and ENM surface charge are key factors influencing protein corona formation [89].

As the fabrication and use of nanoproducts accelerates, more ENMs are being released into the environment at an exponential rate [85]. Compared with the corresponding bulk materials, ENMs possess relatively high surface reactivity and a larger surface area, are abundant in adsorption binding sites, and display unique physicochemical properties [86] (Figure 4a). Surface contact is the first and most important step in the interaction between emitted ENMs and environmental components, and more studies are underway to demonstrate interfacial interactions and their role in the environmental behavior and biological effects of ENMs [85,87]. The inclusion of nano–biomacromolecule and nano–pollutant interactions is a major distinguishing feature of this review. Many nanotoxicity models focus only on pristine material descriptors, but real exposure environments continuously reshape ENMs through corona formation, dissolution, aggregation, and adsorption of coexisting contaminants. Considering these interactions is essential for environmentally realistic and biologically meaningful risk assessments.

### 5.1. Nano–Biomacromolecule Interactions

ENMs can enter the human body or biological system through accidental (breathing, ingestion, skin contact, food chain accumulation) and purposeful (medical and diagnostic) methods. Therefore, the interaction between ENMs and biological systems is inevitable [88] (Figure 4b). In addition to ENMs, there are natural “nanomaterials” in biological systems, such as proteins, DNA molecules, and lipids. These biological molecules constitute the basic unit of life-cells [88]. The interactions between ENMs and different levels of biological systems are based on their interactions with biomolecules. When ENMs come into contact with biological fluids (such as blood, lymph, alveolar fluid), countless biological molecules are adsorbed on their surface to form a “Biocorona” [9]. Therefore, the study of interactions between ENMs and biomolecules is very important for understanding the biological effects of ENMs.

Characterization techniques such as omics techniques, cryo-electron microscopy, high-resolution transmission electron microscopy, and atomic force microscopy can be used for biocorona analysis to obtain information about the arrangement and distribution of biomolecules in the biocorona [90,91]. However, the types of ENMs are very rich; the above-mentioned cutting-edge experimental methods are time-consuming, labor-intensive, and expensive, and it is difficult to comprehensively measure the interactions between ENMs and biomolecules [92,93]. Therefore, researchers have developed a series of prediction models for interactions between ENMs and biomolecules.

Traditional linear regression models have difficulty describing the composition of the biocorona, and ML algorithms can overcome this difficulty. Ban et al. mined and analyzed 652 data related to protein corona on various nanomaterials in 56 studies, established a highly heterogeneous database, and the effect of surface modification of nanoparticles on the composition of protein corona was determined by the RF algorithm [94]. Findlay et al. used ensemble ML to reveal that the key factor influencing protein corona formation is the surface charge of proteins and ENMs [89] (Figure 4c). To address the problem of a lack of descriptors, and inspired by face recognition technology, Yan et al. developed a novel nanostructure annotation method that can automatically convert nanostructures into images for convolutional neural network modeling without the use of complex nanodescriptors to predict the adsorption of nanomaterials to proteins [95].

Aside from proteins, interactions of other biomolecules with ENMs in biocorona prediction simulation studies are also very important for an in-depth understanding of nanobiological effects and, as such, require further attention. In addition, the experimental methods and experimental conditions for qualitative and quantitative analyses of biocorona greatly affect the results; therefore, there is obvious heterogeneity in the data, posing challenges to the establishment of predictive models [96].

Therefore, future ML models should treat the biocorona not as an experimental complication but as a dynamic descriptor of biological identity. This perspective is innovative because it links molecular-level adsorption events with cellular recognition, biodistribution, immune responses, and organ toxicity, thereby strengthening the mechanistic basis of nanomaterial risk assessment.

### 5.2. Nano–Pollutant Interactions

**Figure 5:**
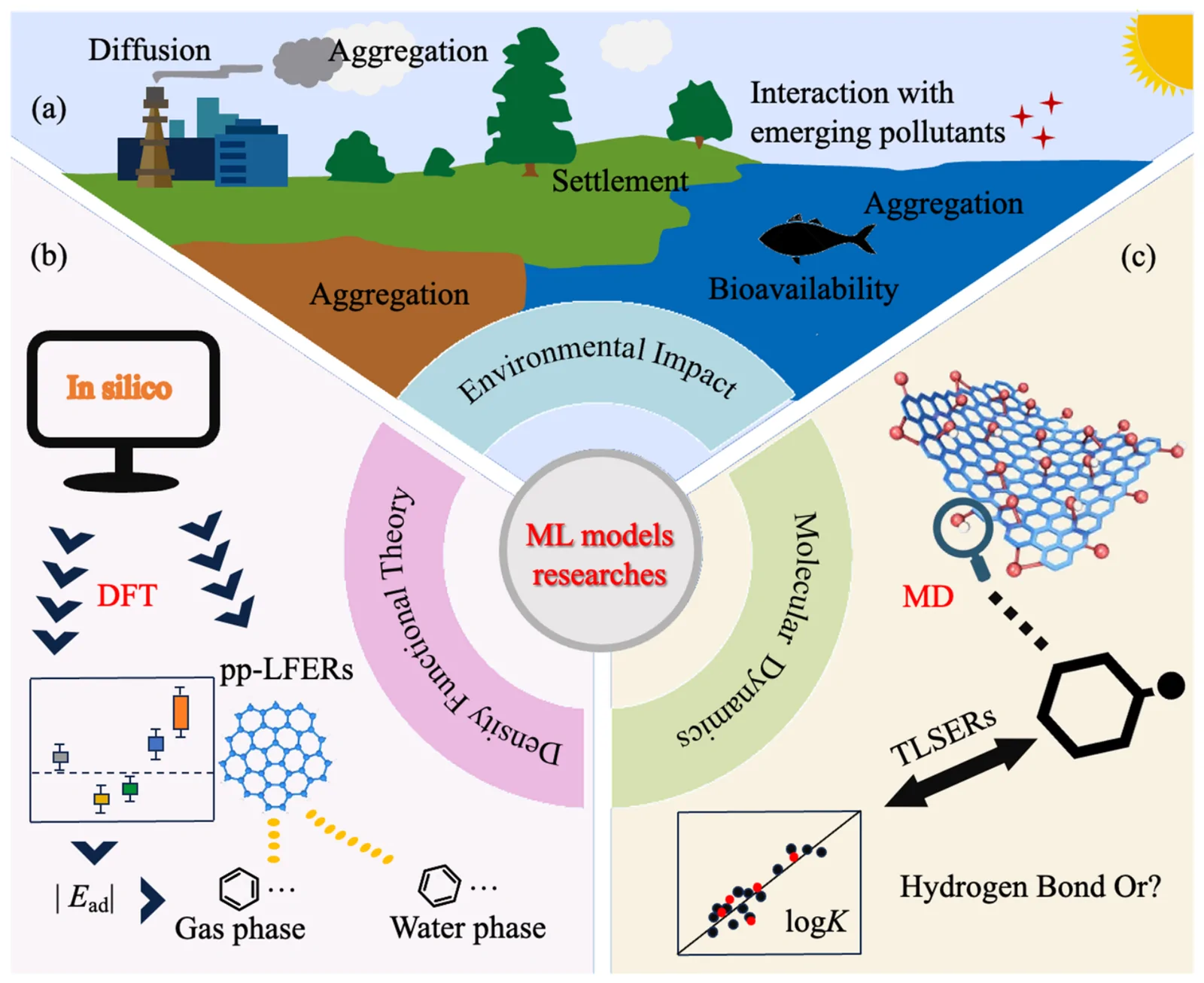
Impacts of ENMs entering the environment: (**a**) ENMs undergo various physicochemical transformations after release, interacting with emerging pollutants in soil and water environments [97]. (**b**) Gas-phase and aqueous-phase adsorption energies of organic pollutants on graphene surfaces can be predicted [100]. (**c**) These adsorption processes were simulated using DFT methods [101].

Once released into the environment, ENMs undergo various physicochemical transformation processes. When ENMs diffuse in water and soil environments, they interact with emerging pollutants in the environment and affect their environmental fate and environmental risks (Figure 5a) [97]. Both traditional experimental methods and modern simulation methods can be used to study the interactions between them. However, experimental evaluation is expensive and time-consuming, and simulation by introducing density functional theory (DFT) or molecular dynamics (MD) methods cannot only avoid these shortcomings but also provide more details at the atomic level. At present, QSAR has been widely used to predict the interaction between nanomaterials and organic pollutants. Here, we will show some models and prediction examples of the interaction between carbon nanomaterials and organic pollutants [98,99].

Wang et al. calculated and simulated the gas-phase and water-phase adsorption of 38 organic compounds (aliphatic compounds, benzene and its derivatives, and polycyclic aromatic hydrocarbons) on graphene surfaces using the DFT method (Figure 5b). Two multi-parameter linear free energy relationships (pp-LFERs) were established, which can be used to predict the adsorption energies of organic compounds on graphene surfaces in gas and aqueous phases and to estimate the contribution of various interactions to adsorption based on the pp-LFER model (Figure 5a). The main driving forces for gas-phase adsorption are dispersion and electrostatic interactions; adsorption in the aqueous phase is mainly affected by dispersibility and hydrophobicity [100]. In addition, the adsorption equilibrium constant (*K*) is the characteristic constant of adsorption reactions. In order to explore the effect of functional groups on adsorption, Wang et al. used MD simulation to predict log*K* values of 43 aromatic compounds in water and graphene with different oxidized functional groups. Two theoretical linear dissolution energy relationship models were constructed to predict the log*K* values of organic matter on the surface of graphene and graphene oxide in water. The model results show that the hydrogen-donating capacity, dispersibility, and hydrophobic interaction have the most significant effects on the log*K* value of the graphene surface; for the log*K* value of the graphene oxide surface, the hydrogen-donating capacity has the most significant effect (Figure 5c) [101]. Encouragingly, significant progress has been made in the field of interactions between ENMs and environmental pollutants. Long et al. [102] advanced the prediction of adsorption mechanisms of heavy metal nanoparticles on carbon-based materials such as activated carbon and biochar. By employing three ML algorithms, including RF, Gradient Boosting Decision Tree (GBDT), and Extreme Gradient Boosting (XGB), in combination with adsorption characteristics, experimental conditions, and the physicochemical properties of heavy metals, the study identified key parameters influencing adsorption affinity. The developed algorithms were further integrated into a graphical user interface software package to facilitate user accessibility and broader dissemination. However, the accurate quantification of adsorption capacity remains an unresolved challenge.

Table 3 compares the principal modeling strategies used for nano–biomacromolecule and nano–pollutant interactions. Protein-corona studies mainly use particle size, surface charge and modification, protein properties, or image-derived features to predict corona composition or adsorption, whereas pollutant interaction studies combine quantum-chemical, molecular dynamics, and experimental descriptors to estimate adsorption energies, equilibrium constants, or affinities [103]. Image-based convolutional models reduce dependence on manually engineered descriptors, while DFT- and MD-informed models offer greater physicochemical interpretability; however, each approach remains sensitive to the composition of the biological or environmental medium. Across both application areas, inconsistent endpoint definitions, limited sample diversity, and heterogeneous validation strategies constrain direct comparison and transferability. These patterns indicate that future models should combine standardized interfacial descriptors with external validation across media, nanomaterial classes, and laboratories [12]. Systematic adsorption prediction studies for organic compounds remain scarce across many emerging nanomaterial classes, and existing frameworks are insufficient to capture the multidimensional nature of pollutant–nanomaterial interfacial processes. Furthermore, ENMs do not exist in isolation within environmental systems; they may function as carriers, sinks, catalysts, or transformation platforms for co-occurring contaminants, rendering their risks inextricable from the surrounding chemical matrix. Consequently, ML models that integrate pollutant properties, nanomaterial surface chemistry, medium composition, and adsorption mechanisms into a unified predictive framework are urgently needed to enable comprehensive assessment of combined environmental and health risks.

With the rapid improvement of computing power, great breakthroughs will be made in nano-molecular interaction research based on ML and molecular simulation in the future, providing more possibilities for the safe and effective application of ENMs. By integrating molecular simulation with ML, future studies can move from an empirical association toward mechanistic prediction of nano-interface processes. This integration represents an important frontier because it can generate physically interpretable descriptors, reduce experimental workloads, and improve the transferability of models across nanomaterial classes and exposure scenarios [104].

## 6. Conclusions and Perspectives

Across the reviewed literature, a common limitation is the reliance on relatively small and heterogeneous datasets assembled from individual laboratories or published studies, with inconsistent reporting of nanomaterial characterization, dose metrics, exposure durations, biological systems, and endpoint definitions. Dataset reliability depends not only on the sample size but also on whether the tested nanomaterial is adequately characterized in the relevant exposure medium. Reporting nominal primary particle size alone is insufficient because size distribution, shape or aspect ratio, surface chemistry, coating identity and density, surface charge, dissolution, aggregation/agglomeration state, and medium-dependent corona formation can alter the biological identity of an ENM. Missing or non-harmonized descriptors may introduce hidden confounding, causing a model to learn laboratory- or protocol-specific patterns rather than transferable toxicity relationships. Future databases should therefore distinguish pristine, administered, and biologically transformed material states, report characterization methods and measurement uncertainty, and explicitly identify missing values. When imputation is unavoidable, it should be accompanied by missingness indicators and sensitivity analyses.

Data coverage is also uneven: pulmonary cytotoxicity and inflammation are comparatively well represented, whereas extrapulmonary toxicity, biomolecular corona formation, and nano–pollutant co-exposure remain data-sparse and material-specific. Consequently, the performance metrics reported by different studies are not directly comparable. Descriptor-based QSAR/QNAR, random forest, and support vector approaches are generally suitable for structured datasets and can provide useful feature rankings, whereas neural networks, image-based convolutional models, and hybrid simulation–ML approaches can capture more complex non-linear or descriptor-free relationships but require larger datasets and more rigorous external validation. Although nested cross-validation and IVIVE-oriented modeling represent important methodological advances, random record-level splitting may overestimate performance when closely related observations from the same study or nanomaterial occur in both the training and test sets. Validation that excludes complete studies or nanomaterial classes therefore remains an important unmet need.

Biological and regulatory relevance also varies substantially among model types. Models trained primarily on cell-viability endpoints are useful for initial hazard screening but cannot fully represent biodistribution, chronic exposure, organ-specific mechanisms, dynamic biomolecular corona formation, or mixture effects. Mechanistically informed descriptors, multi-organ endpoints, and IVIVE frameworks improve biological plausibility; nevertheless, algorithmic feature importance should not be interpreted as causal evidence without experimental confirmation. For regulatory application, predictions should be accompanied by transparent data provenance, standardized reporting, defined applicability domains, uncertainty estimates, reproducible workflows, and independent or prospective validation. Future studies should therefore prioritize curated and interoperable databases, harmonized dose and endpoint reporting, study-wise and material-wise validation, explainable models linked to adverse mechanisms, and integration into tiered testing and SSbD frameworks in which targeted in vivo testing is reserved for uncertain or out-of-domain cases.

Within the European Union, the Registration, Evaluation, Authorisation and Restriction of Chemicals (REACH) framework includes nanoform-specific information requirements, while the Classification, Labeling and Packaging (CLP) regulation covers nanoforms, even though it does not establish a separate nanomaterial definition [105]. For nanomaterials, chemical composition alone is insufficient to define regulatory identity or similarity: particle-size distribution, shape and aspect ratio, surface chemistry and coating, dissolution, aggregation/agglomeration, and transformations in the relevant exposure medium can change hazard and exposure profiles. Machine learning-supported grouping, read-across, and data-gap filling should therefore be applied only within clearly justified nanoform boundaries and should report data provenance, endpoint relevance, applicability domains, and predictive uncertainty. Such predictions are most defensible as one component of a weight-of-evidence assessment rather than as stand-alone regulatory evidence [105].

The broader SSbD framework provides the more appropriate design perspective for this field. It extends earlier hazard-centered safety-by-design concepts by evaluating safety together with functionality, exposure, resource and energy use, releases across the life cycle, persistence, durability, recyclability, and end-of-life impacts. In line with the revised European SSbD framework, multi-objective ML could compare candidate nanoforms across these dimensions, identify trade-offs early in development, and direct experimental resources toward the most informative or uncertain design options [106,107,108]. SSbD is a voluntary innovation framework that can promote anticipatory decisions beyond minimum legal compliance, but it does not replace the obligations established by REACH, CLP, or other sector-specific legislation [106].

Regulatory translation also depends on data and mechanistic organization. Applying the Findable, Accessible, Interoperable, and Reusable (FAIR) principles to nanosafety data requires persistent identifiers, machine-readable metadata, shared vocabularies, traceable protocols, and explicit links between pristine, administered, and transformed material states [109,110]. Recent nanosafety-specific FAIR assessments show that inconsistent reporting and missing metadata remain major barriers to reuse and computational risk assessments [109]. In parallel, Adverse Outcome Pathways (AOPs) can organize molecular initiating events, key events, and adverse outcomes into mechanistically plausible sequences for nanomaterial grouping and risk assessments [111]. Combining FAIR data, AOP-guided endpoints, explainable ML, and study-wise or material-wise external validation would make predictions more reusable, biologically interpretable, and suitable for tiered regulatory decision-making.

Overall, this review demonstrates that ML is becoming a necessary component of modern nanosafety assessment because it can address four persistent bottlenecks: limited animal testing capacity, fragmented and heterogeneous literature data, weak mechanistic interpretation of complex nano–bio interactions, and insufficient translation from laboratory assays to regulatory decision-making. The novelty of the review lies in its integrated framework connecting organ-specific toxicity, nano–biomacromolecule interactions, nano–pollutant interactions, IVIVE, interpretability, and SSbD strategies. ML should be positioned as a decision-support component of a tiered testing strategy rather than as a complete substitute for animal experiments. ML models can first screen large ENM libraries, rank materials by predicted hazard, identify major toxicity-driving factors, and extrapolate in vitro responses to in vivo outcomes [7,33,34]. Animal testing can then be restricted to materials outside the model’s applicability domain, predictions with high uncertainty, novel mechanisms or exposure scenarios, and confirmatory endpoints required for regulatory decisions. Active learning can further identify the smallest and most informative set of new experiments, whose results can be fed back into the model to progressively reduce repetitive or low-information animal testing.

Existing animal test data provide valuable training information but must first be curated and standardized. In supervised learning, in vivo outcomes can serve as target labels, while nanomaterial composition, size, shape, surface chemistry, dose, exposure route and duration, species, target organ, and corresponding in vitro or omics measurements serve as input features. Multi-task learning, transfer learning, and IVIVE frameworks can then identify shared toxicity patterns across materials, species, and endpoints [19,33,34,69,81]. Study-level quality and uncertainty should be encoded, and validation should separate entire studies or nanomaterial types to prevent data leakage and determine when predictions are sufficiently reliable and when targeted in vivo confirmation remains necessary.

## Figures and Tables

**Table 2:** ML models for extrapulmonary organ toxicity of nanomaterials.

Nanomaterials	Organism/Organ/Cell	Key Descriptors	Endpoint	Reference
Ag, TiO_2_ nanoparticles	Rat BRL 3A hepatic cells	Dose, enthalpy of formation, exposure time, hydrodynamic size	Hepatotoxicity: cytotoxicity, mitochondrial dysfunction, oxidative stress	[50]
7 oxide nanomaterials	Human L-02, Chang-Liver hepatocytes	Dose, enthalpy of formation, exposure time	Liver injury classification	[52]
20 nm and 50 nm SiO_2_ nanoparticles	HEK293 human embryonic kidney cells	Particle size, concentration, exposure time (0–48 h)	Dose-dependent cell viability	[55]
Nano-TiO_2_ mixed with heavy metals	HK-2 human renal tubular cells	Nanomaterial concentration, heavy metal ratio, exposure time	Nephrotoxicity, cell viability, ROS generation	[56]
ZnO nanoparticles	Rat kidney tissue, human renal epithelial cells	Particle size, dissolution rate, lipid species composition	Mitochondrial/membrane damage, lipid metabolic disorder	[53,54]
Metal oxide nanoparticles	Zebrafish (*Danio rerio*) embryos	Core element, shell ligand, particle size	Embryonic lethal reproductive/developmental toxicity	[72]
18+ diverse ENMs	Rat reproductive tissue	Element composition, exposure route	Gonadal accumulation, reproductive toxicity risk	[69]
SWCNTs, fullerenes, CdSe QDs, carbon black	Rat PC-12 neuronal cells	Particle concentration, surface coating	SBDP production, cell vacuolation	[79]
CdSe quantum dots	Primary rat hippocampal neurons	Nanoparticle concentration	Cytoplasmic Ca^2+^ overload, neuronal apoptosis	[80]
Various carbon/metal/oxide ENMs	In vitro cell lines + rodent multi-organ tissue	Physicochemical features, exposure route	Multi-organ hazard ranking	[81]

**Table 3:** ML and simulation models for nano–biomacromolecule and nano–pollutant interactions.

Nanomaterial/System	Matrix/Medium	Key Descriptors	Endpoint	Reference
Diverse ENMs (metal, metal oxide, carbon nanomaterials)	Plasma protein mixture	Nanoparticle size, surface charge, surface modification, protein isoelectric point	Protein corona composition prediction	[94]
Silver nanoparticles	Single protein solution	NP surface potential, protein hydrophobicity, molecular weight	Protein adsorption quantity	[89]
Various ENMs	Biological fluid image dataset	2D nanostructure pixel features (no manual descriptors)	Protein adsorption via CNN model	[95]
Graphene, graphene oxide	Aqueous phase	DFT energy parameters, molecular polarity, dispersion forces	Organic pollutant adsorption energy	[100]
Oxidized graphene variants	Aqueous solution	Oxidation degree, hydrogen bonding intensity, hydrophobic factor	Adsorption equilibrium constant logK	[101]
Activated carbon, MWCNTs, biochar	Soil and water matrix	Pore size, metal ion radius, pH, temperature	Heavy metal adsorption affinity	[102]

CNNL: convolutional neural network; DFT: density functional theory; RF: random forest; GBDT: Gradient Boosting Decision Tree; XGBoost: Extreme Gradient Boosting; logK: adsorption equilibrium constant.

## Data Availability

The supporting data or code have been included in the article’s main text.

## Abbreviations

The following abbreviations are used in this manuscript:

A549	Human lung adenocarcinoma
AI	Artificial intelligence
AOPs	Adverse outcome pathways
BEAS-2B	Human lung cells
BN	Bayesian network
BTB	Blood–testis barrier
CLP	Classification, Labeling and Packaging Regulation
CNS	Central nervous system
DFT	Density functional theory
ENMs	Engineered nanomaterials
IVIVE	In vitro-to-in vivo extrapolation
GNP	Gold nanoparticle
IL-1β	Pro-inflammatory cytokine
LC_50_	Median lethal concentration
MD	Molecular dynamics
MeONPs	Metal oxide nanoparticles
ML	Machine learning
NPs	Nanoparticles
PLS	Multiple partial least squares regression
pp-LFERs	Two multi-parameter linear free energy relationships
QD	Quantum dot
QNAR	Quantitative nanostructure activity relationship
QSARs	Quantitative structure-activity relationships
REACH	Registration, Evaluation, Authorisation and Restriction of Chemicals
RF	Random forest regression
ROS	Reactive oxygen species
SBDP	αII-spectrin breakdown products
SSbD	Safe-and-sustainable-by-design
SWNTs	Single-walled nanoparticles
SWCNTs	Single-walled carbon nanotubes
TBRFA	Feature interaction network analysis framework
vGNP	Virtual gold nanoparticle
